# Protective Effects of 6-Shogaol, an Active Compound of Ginger, in a Murine Model of Cisplatin-Induced Acute Kidney Injury

**DOI:** 10.3390/molecules26195931

**Published:** 2021-09-30

**Authors:** Mi-Gyeong Gwon, Hyemin Gu, Jaechan Leem, Kwan-Kyu Park

**Affiliations:** 1 Department of Pathology, School of Medicine, Catholic University of Daegu, Daegu 42472, Korea; daldy88@cu.ac.kr (M.-G.G.); guhm1207@cu.ac.kr (H.G.); kkpark@cu.ac.kr (K.-K.P.); 2 Department of Immunology, School of Medicine, Catholic University of Daegu, Daegu 42472, Korea

**Keywords:** 6-shogaol, cisplatin, acute kidney injury, oxidative stress, apoptosis, necroptosis, inflammation

## Abstract

Acute kidney injury (AKI) is a dose-limiting side effect of cisplatin therapy in cancer patients. However, effective therapies for cisplatin-induced AKI are not available. Oxidative stress, tubular cell death, and inflammation are known to be the major pathological processes of the disease. 6-Shogaol is a major component of ginger and exhibits anti-oxidative and anti-inflammatory effects. Accumulating evidence suggest that 6-shogaol may serve as a potential therapeutic agent for various inflammatory diseases. However, whether 6-shogaol exerts a protective effect on cisplatin-induced renal side effect has not yet been determined. The aim of this study was to evaluate the effect of 6-shogaol on cisplatin-induced AKI and to investigate its underlying mechanisms. An administration of 6-shogaol after cisplatin treatment ameliorated renal dysfunction and tubular injury, as shown by a reduction in serum levels of creatinine and blood urea nitrogen and an improvement in histological abnormalities. Mechanistically, 6-shogaol attenuated cisplatin-induced oxidative stress and modulated the renal expression of prooxidant and antioxidant enzymes. Apoptosis and necroptosis induced by cisplatin were also suppressed by 6-shogaol. Moreover, 6-shogaol inhibited cisplatin-induced cytokine production and immune cell infiltration. These results suggest that 6-shogaol exhibits therapeutic effects against cisplatin-induced AKI via the suppression of oxidative stress, tubular cell death, and inflammation.

## 1. Introduction

Acute kidney injury (AKI) is defined as a sudden decrease in kidney function and is strongly associated with increased morbidity and mortality in hospitalized patients [1]. Furthermore, accumulating evidence suggest that AKI is significantly linked to an increased risk of chronic kidney disease [2]. There are many possible causes of AKI, such as sepsis, volume depletion, and nephrotoxic medications [1]. Among them, nephrotoxic mediation-associated AKI accounts for 19–26% of AKI cases among hospitalized patients [3]. Over the decades, many chemotherapeutic agents have been developed for cancer patients, but their use is frequently limited by serious side effects, including nephrotoxicity [4]. Cisplatin is one of the most effective chemotherapeutic agents and is widely used in the treatment of various solid tumors, including lung, testicular, ovarian, and bladder cancers [5]. However, nephrotoxicity is a major side effect of cisplatin-based therapies, with a 20–35% risk in patients receiving cisplatin, limiting the application and efficacy of cisplatin in cancer treatment [6]. Because there is no validated pharmacological treatment for cisplatin-induced AKI, the development of novel medications for the renal side effect of cisplatin-based therapies is urgently needed. Although the pathogenesis of cisplatin-induced AKI is still incompletely understood, multiple mechanisms, such as oxidative stress, tubular cell death, and inflammation, are involved in the pathological process [5,7]. Accumulating evidence suggest that many compounds isolated from natural products display potent anti-oxidative and anti-inflammatory activities [8]. Thus, natural products can serve as promising sources of lead compounds to achieve the discovery and development of new drugs for various human diseases [9]. Indeed, in recent studies, many natural product-derived compounds, such as curcumin, quercetin, luteolin, and berberine, have been reported to have preventive or therapeutic effects on cisplatin-induced AKI [6].

Ginger is a well-known herbaceous plant and has long been used for relieving motion sickness, nausea, and abdominal pain [10]. It is known that ginger contains various bioactive compounds such as terpene, phenolic, and aromatic compounds [11]. Among them, 6-shogaol is a main bioactive phenolic compound of dried ginger and exerts potent anti-oxidative and anti-inflammatory effects [10]. Previous studies have shown that an administration of 6-shogaol to rodents ameliorated various inflammatory diseases [10]. However, whether 6-shogaol has a beneficial effect on cisplatin-induced AKI has not yet been determined. Therefore, in the present study, we evaluated the effect of 6-shogaol on cisplatin-induced AKI and explored the underlying mechanisms.

## 2. Results

### 2.1. 6-Shogaol Ameliorated Cisplatin-Induced AKI

**Figure 1:**
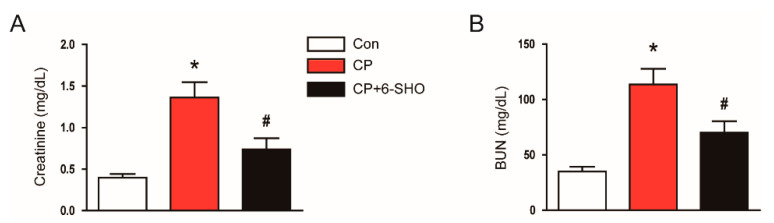
Effect of 6-shogaol on renal function in cisplatin-treated mice. Mice were given an intraperitoneal injection of 6-shogaol (20 mg/kg; 6-SHO) daily for 3 consecutive days, starting from 1 h after cisplatin treatment (20 mg/kg; CP). All mice were sacrificed 72 h after a single dose of cisplatin and blood samples were obtained. (**A**) Serum creatinine levels. (**B**) Blood urea nitrogen (BUN) levels. *n* = 8 per group of mice. * *p* < 0.05 versus the control group (Con). ^#^ *p* < 0.05 versus the cisplatin-treated group (CP).

To evaluate the effect of 6-shogaol on renal function, we measured serum levels of creatinine and blood urea nitrogen (BUN), indicators of renal function [12], in all three groups of mice. A dose of 20 mg/kg cisplatin markedly increased the levels of both markers (Figure 1A,B). However, the administration of 6-shogaol after cisplatin treatment significantly attenuated the cisplatin-induced renal dysfunction (Figure 1A,B).

**Figure 2:**
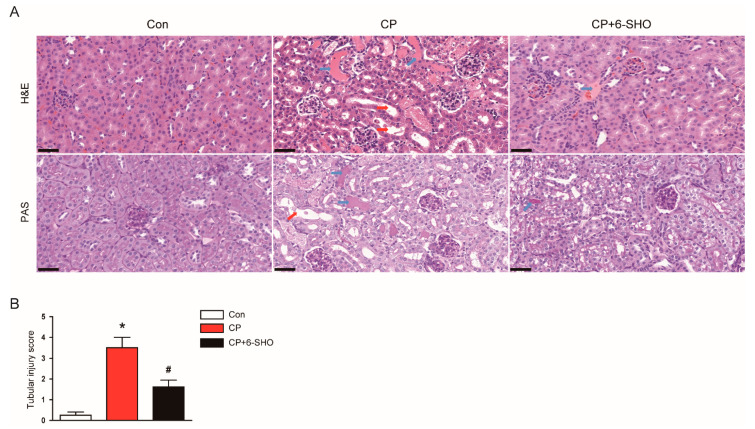
Effect of 6-shogaol on histological abnormalities in cisplatin-treated mice. (**A**) Representative images of hematoxylin and eosin (H&E) or periodic acid-Schiff (PAS) staining of renal cortex. Scale bar = 40 μm. Red arrows indicate tubular dilatation. Blue arrows indicate cast deposition in the lumens of tubules. (**B**) Tubular injury was semiquantitatively scored using PAS-stained sections. *n* = 8 per group of mice. * *p* < 0.05 versus Con. ^#^ *p* < 0.05 versus CP.

Because tubular injury is a hallmark of cisplatin-induced AKI [13], we next performed a histological examination of the kidneys. Hematoxylin and eosin (H&E) and periodic acid-Schiff (PAS) staining showed significant tubular damage in cisplatin-treated mice, including tubular dilatation and cast formation (Figure 2A,B). These histological abnormalities were significantly alleviated by 6-shogaol (Figure 2A,B).

**Figure 3:**
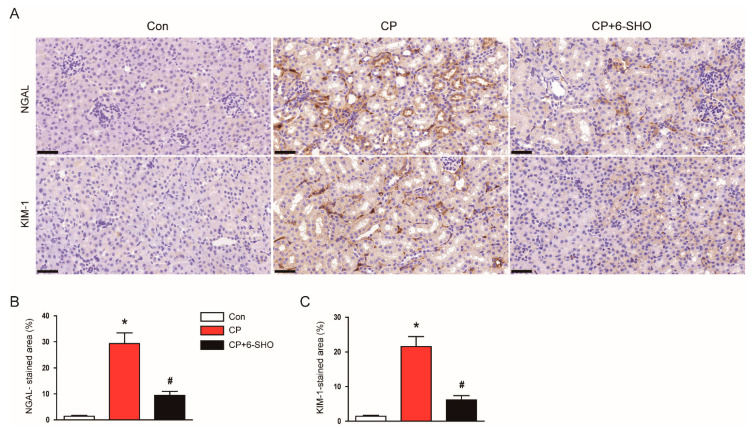
Effect of 6-shogaol on expression of neutrophil gelatinase-associated lipocalin (NGAL) and kidney injury molecule-1 (KIM-1) in cisplatin-treated mice. (**A**) Representative images of immunohistochemistry (IHC) staining for NGAL and KIM-1. Scale bar = 40 μm. (**B**) Quantification of positive staining for NGAL. (**C**) Quantification of positive staining for KIM-1. *n* = 8 per group of mice. * *p* < 0.05 versus Con. ^#^ *p* < 0.05 versus CP.

Neutrophil gelatinase-associated lipocalin (NGAL) and kidney injury molecule-1 (KIM-1) are well-known markers of tubular injury [14]. It has been known that cisplatin treatment markedly increases the renal expression of both markers in rodents [15,16]. Consistently, immunohistochemistry (IHC) staining revealed that cisplatin-treated mice exhibited increased expression of NGAL and KIM-1 (Figure 3A–C). However, the administration of 6-shogaol significantly reduced the expression of both markers (Figure 3A–C). Taken together, these results suggest that 6-shogaol protected mice from cisplatin-induced AKI, as represented by the amelioration of renal dysfunction and histopathological abnormalities.

### 2.2. 6-Shogaol Attenuated Cisplatin-Induced Oxidative Stress

**Figure 4:**
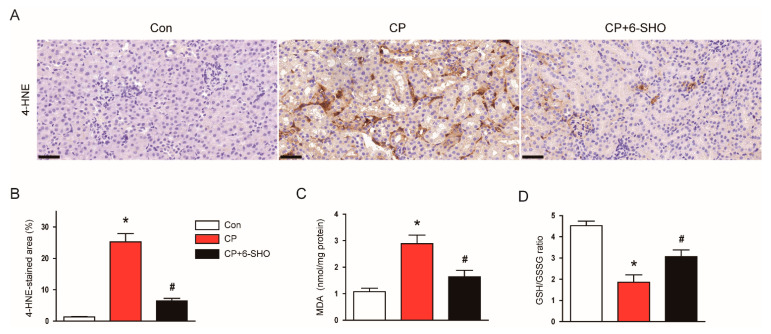
Effect of 6-shogaol on oxidative stress in cisplatin-treated mice. (**A**) Representative images of IHC staining for 4-hydroxynonenal (4-HNE). Scale bar = 40 μm. (**B**) Quantification of positive staining for 4-HNE. (**C**) Renal levels of malondialdehyde (MDA). (**D**) Ratio of reduced glutathione (GSH) to oxidized glutathione (GSSG). *n* = 8 per group of mice. * *p* < 0.05 versus Con. ^#^ *p* < 0.05 versus CP.

Oxidative stress plays a critical role in cisplatin-induced renal injury [5,7]. Previous studies have demonstrated that 6-shogaol exerts an anti-oxidative activity [17,18]. Thus, to explore the mechanisms underlying the protective effect of 6-shogaol on cisplatin-induced AKI, we next evaluated the levels of lipid peroxidation markers 4-hydroxynonenal (4-HNE) and malondialdehyde (MDA) [19] in the kidneys. IHC staining showed that cisplatin treatment largely increased the renal expression of 4-HNE (Figure 4A,B). In addition, renal levels of MDA were elevated after cisplatin treatment (Figure 4C). The increased oxidative stress after cisplatin treatment was also confirmed by a decrease in the ratio of reduced glutathione (GSH) to oxidized glutathione (GSSG) in the kidneys (Figure 4D). However, all these changes were significantly reversed by 6-shogaol (Figure 4A–D).

**Figure 5:**
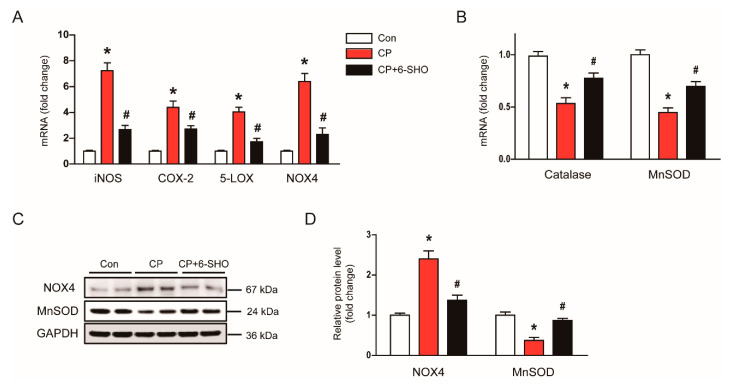
Effect of 6-shogaol on expression of prooxidant and antioxidant enzymes in cisplatin-treated mice. (**A**) Relative mRNA levels of inducible nitric oxide synthase (iNOS), cyclooxygenase-2 (COX-2), 5-lipoxygenase (5-LOX), and nicotinamide adenine dinucleotide phosphate oxidase 4 (NOX4). (**B**) Relative mRNA levels of catalase and manganese superoxide dismutase (MnSOD). (**C**) Western blotting of NOX4 and MnSOD. (**D**) Quantification of western blots for NOX4 and MnSOD. Glyceraldehyde-3-phosphate dehydrogenase (GAPDH) was chosen as an internal control. *n* = 8 per group of mice. * *p* < 0.05 versus Con. ^#^ *p* < 0.05 versus CP.

Previous studies have shown that prooxidant and antioxidant enzymes play important roles in regulating oxidative stress in cisplatin-induced AKI [20,21]. Thus, we examined the effect of 6-shogaol on the expressions of prooxidant and antioxidant enzymes. In cisplatin-treated mice, the mRNA levels of inducible nitric oxide synthase (iNOS), cyclooxygenase-2 (COX-2), 5-lipoxygenase (5-LOX), and nicotinamide adenine dinucleotide phosphate 4 (NOX4) were elevated (Figure 5A), while the expression of catalase and manganese superoxide dismutase (MnSOD) was decreased (Figure 5B). Increased protein levels of NOX4 and MnSOD were also confirmed by western blot analysis (Figure 5C,D). Importantly, the administration of 6-shogaol significantly revered the changes in expression of proxoidant and antioxidant enzymes induced by cisplatin (Figure 5A–D). Altogether, these results suggest that 6-shogaol attenuated the cisplatin-induced oxidative stress, at least in part, through modulating prooxidant and antioxidant systems.

### 2.3. 6-Shogaol Inhibited Cisplatin-Induced Tubular Cell Death

**Figure 6:**
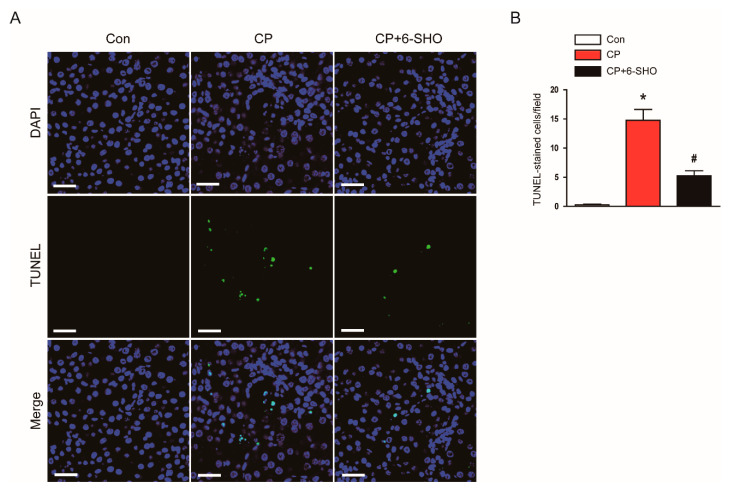
Effect of 6-shogaol on tubular cell apoptosis in cisplatin-treated mice. (**A**) TdT-mediated dUTP nick end labeling (TUNEL) assay on kidney sections. Scale bar = 10 μm. To detect nuclei, 4′, 6-diamidino-2-phenylindole (DAPI) was used. (**B**) Number of TUNEL-stained cells per field. *n* = 8 per group of mice. * *p* < 0.05 versus Con. ^#^ *p* < 0.05 versus CP.

Apoptotic death of tubular epithelial cells is a characteristic feature of cisplatin-induced AKI [5,7]. To evaluate the effect of 6-shogaol on cisplatin-induced tubular cell apoptosis, TdT-mediated dUTP nick end labeling (TUNEL) staining was performed on kidney sections. Cisplatin treatment markedly increased the number of TUNEL-stained cells in the kidneys (Figure 6A,B). However, the administration of 6-shogaol significantly reduced the number of TUNEL-stained apoptotic cells in cisplatin-treated mice (Figure 6A,B).

**Figure 7:**
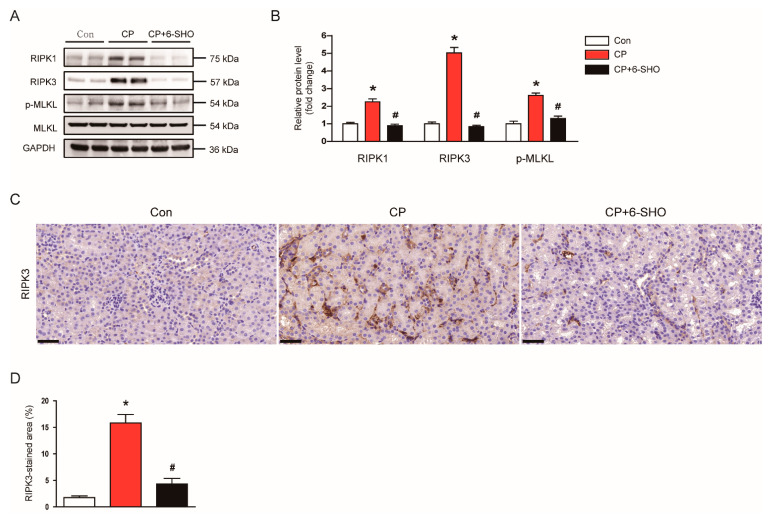
Effect of 6-shogaol on tubular cell necroptosis in cisplatin-treated mice. (**A**) Western blotting of receptor-interacting serine/threonine protein kinase 1 (RIPK1), RIPK3, mixed-lineage kinase domain-like protein (MLKL), and p-MLKL. (**B**) Quantification of western blots for RIPK1, RIPK3, and p-MLKL. GAPDH was used as an internal control. (**C**) Representative images of IHC staining for RIPK3. Scale bar = 40 μm. (**D**) Quantification of positive staining for RIPK3. *n* = 8 per group of mice. * *p* < 0.05 versus Con. ^#^ *p* < 0.05 versus CP.

Recent studies have shown that necroptosis, a programed form of necrosis, is also critically involved in the pathogenesis of cisplatin-induced AKI [22,23]. Because necroptosis is regulated by receptor-interacting serine/threonine protein kinase 1 (RIPK1), RIPK3, and mixed lineage kinase domain-like protein (MLKL), we examined the effect of 6-shogaol on RIPK1-RIPK3-MLKL signaling cascade. Cisplatin-treated mice exhibited increased protein levels of RIPK1, RIPK3, and p-MLKL in kidneys (Figure 7A,B). An upregulation of RIPK3 after cisplatin treatment was also confirmed by IHC staining (Figure 7C,D). Importantly, the administration of 6-shogaol significantly inhibited the cisplatin-induced necroptosis (Figure 7A–D).

### 2.4. 6-Shogaol Suppressed Cisplatin-Induced Inflammation

**Figure 8:**
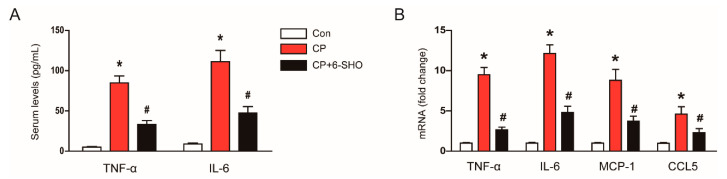
Effect of 6-shogaol on production of cytokines and chemokines in cisplatin-treated mice. (**A**) Serum levels of tumor necrosis factor-α (TNF-α) and interleukin-6 (IL-6). (**B**) Relative mRNA levels of TNF-α, IL-6, monocyte chemoattractant protein-1 (MCP-1), and C-C motif chemokine ligand 5 (CCL5). GAPDH was chosen as an internal control. *n* = 8 per group of mice. * *p* < 0.05 versus Con. ^#^ *p* < 0.05 versus CP.

It has been known that cisplatin treatment induces a significant production of inflammatory cytokines and chemokines and an infiltration of immune cells into the kidney [24,25]. Because 6-shogal has been shown to exhibit anti-inflammatory activity [10], we evaluated whether 6-shogaol can suppress inflammatory responses induced by cisplatin. Cisplatin treatment increased serum levels of tumor necrosis factor-α (TNF-α) and interleukin-6 (IL-6) (Figure 8A). Renal mRNA levels of TNF-α, IL-6, monocyte chemoattractant protein-1 (MCP-1), and C-C motif chemokine ligand 5 (CCL5) were also elevated after cisplatin treatment (Figure 8B). However, the excessive production of cytokines and chemokines induced by cisplatin was significantly suppressed by 6-shogaol (Figure 8A,B).

**Figure 9:**
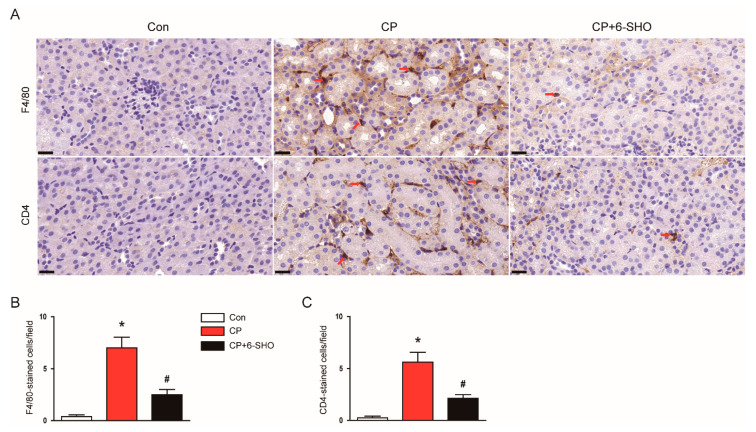
Effect of 6-shogaol on immune cell infiltration in cisplatin-treated mice. (**A**) Representative images of IHC staining for F4/80 and CD4. Red arrows indicate positively stained cells. Scale bar = 20 μm. (**B**) Number of F4/80-stained cells per field. (**C**) Number of CD4-stained cells per field. *n* = 8 per group of mice. * *p* < 0.05 versus Con. ^#^ *p* < 0.05 versus CP.

We next examined the effect of 6-shogaol on the infiltration of macrophages and CD4^+^ T cells into the kidney. IHC staining of the kidney sections showed that the number of cells stained with F4/80 or CD4 was increased after cisplatin treatment (Figure 9A–C). However, the administration of 6-shogaol significantly suppressed the infiltration of these cells (Figure 9A–C).

## 3. Discussion

In this study, we aimed to evaluate the effect of 6-shogaol on cisplatin-induced AKI. Our data showed that the administration of 6-shogaol after cisplatin treatment attenuated cisplatin-induced renal dysfunction and tubular injury. These therapeutic effects of 6-shogaol were associated with an amelioration of oxidative stress, tubular cell death, and inflammation.

Ginger has been widely used as a medicinal herb, especially in Asia [10]. Among the components of ginger, 6-gingerol is the most bioactive component in fresh ginger, whereas 6-shogaol is the main bioactive compound in dried ginger [10]. Accumulating evidence suggest that 6-shogaol exerts beneficial effects against various inflammatory diseases such as periodontitis [26], endometriosis [27], diabetic neuropathy [28], multiple sclerosis [29], Parkinson’s disease [30], asthma [31], and allergic dermatitis [32]. Importantly, a recent study showed that an administration of 6-shogaol protected against renal ischemia-reperfusion injury in mice [33]. Ischemia-reperfusion injury is one of the most common causes of AKI and is closely associated with high morbidity and mortality [34]. In this study, we demonstrated that 6-shogaol had a protective effect on cisplatin-induced AKI. Because nephrotoxic medication-associated AKI is also a major cause of AKI [3], these results suggest that 6-shogaol has the potential to protect against AKI caused by various etiologies. Furthermore, 6-shogaol has been shown to exert a protective effect against diabetic nephropathy, the most common cause of chronic kidney disease in mice [35,36].

Although the mechanism of cisplatin-induced AKI has not been fully elucidated despite many efforts, oxidative stress is believed to play an important role in the pathophysiology of cisplatin-induced AKI [5,7]. Indeed, many studies have examined the use of antioxidants for the prevention or treatment of cisplatin-induced renal injury [37]. A previous study showed that 6-shogaol has an antioxidant property and its antioxidant activity is stronger than that of 6-gingerol [38]. These results prompted us to examine the effect of 6-shogaol on oxidative stress in cisplatin-induced renal injury. The administration of 6-shogaol suppressed cisplatin-induced oxidative stress, as shown by a decrease in the amount of lipid peroxidation by-products. In addition, cisplatin treatment decreased the GSH/GSSG ratio, which was significantly reversed by 6-shogaol. A decrease in the GSH/GSSG ratio indicates increased oxidative stress [39,40]. Altogether, our data indicates that 6-shogaol exhibited an anti-oxidative effect in cisplatin-induced AKI. Consistently, the protective effects of 6-shogaol on diabetic nephropathy were associated with a suppression of oxidative stress [35,36]. A recent study also reported that pretreatment of 6-shogaol inhibited oxidative stress to ameliorate middle cerebral artery occlusion-induced brain damage [41]. It has been known that oxidative stress occurs due to an imbalance between prooxidant and antioxidant systems [42]. In cisplatin-induced AKI, the renal expression of prooxidant enzymes, such as iNOS, COX-2, 5-LOX, and NOX4, was increased [21,25], while that of antioxidant enzymes, such as catalase and MnSOD [19], was decreased. Our data suggest that 6-shogaol modulated prooxidant and antioxidant systems to inhibit cisplatin-induced oxidative stress. The nuclear factor erythroid 2-related factor 2 (Nrf2) is a critical transcription factor that regulates the expression of antioxidant enzymes [43]. The expression of catalase and MnSOD can be regulated by Nrf2. Recent studies have shown that 6-shogaol activated Nrf2 to upregulate antioxidant enzymes, resulting in the suppression of oxidative stress [17,44].

Tubular cell death is a hallmark of cisplatin-induced AKI [5,7]. The administration of 6-shogaol largely reduced cisplatin-induced apoptosis, as shown by a decrease in TUNEL-stained cells. In agreement with our data, it was reported that 6-shogaol treatment reduced tubular cell apoptosis in a murine model of ischemic AKI [33]. In cisplatin-induced AKI, oxidative stress can lead to tubular cell apoptosis [45]. Thus, the suppression of oxidative stress by 6-shogaol may have induced a decrease in apoptotic death. According to the results of recent studies, besides apoptosis, necroptosis is known to play an important role in cisplatin-induced AKI [22,23]. During necroptosis, RIPK1 interacts with RIPK3 to form a heterodimer complex, promoting the oligomerization of MLKL by phosphorylating it [34]. The oligomeric form of MLKL translocates to the plasma membrane, resulting in membrane rupture. Mice deficient for RIPK3 or MLKL exhibited less severe renal injury after cisplatin treatment compared to wild-type mice [22]. A pharmacological suppression of RIPK1 activity ameliorated tubular cell necroptosis and renal injury in cisplatin-treated mice [46,47]. Previous studies have reported that the renal expression of RIPK1, RIPK3, and p-MLKL was increased after cisplatin treatment [48,49]. Interestingly, these changes were significantly attenuated by 6-shogaol. Altogether, these data indicate that 6-shogaol inhibited two major types of tubular cell death, apoptosis and necroptosis, in cisplatin-induced renal injury.

It has been known that inflammation is critically involved in cisplatin-induced AKI [5,7]. Cisplatin treatment induces systemic and renal inflammation and recruits immune cells into the kidneys, promoting the production of more inflammatory cytokines and chemokines, leading to severe kidney damage. In the present study, we found that 6-shogaol reduced serum and renal levels of TNF-α and IL-6, suggesting the inhibitory effect of 6-shogaol on systemic and renal inflammation. In particular, the importance of TNF-α in the pathogenesis of cisplatin-induced AKI has been well elucidated in previous studies [5,7]. Mice deficient for TNF-α were resistant to kidney damage induced by cisplatin [50,51]. A pharmacological inhibition of TNF-α blunted cisplatin-induced production of other cytokines and chemokines, and attenuated cisplatin-induced renal injury [50]. In addition, the administration of 6-shogaol also reduced the elevated expression of chemokines, MCP-1 and CCL5. These chemokines play important roles in recruiting macrophages and T cells into the tissues [52]. In the present study, an increased infiltration of macrophages and CD4^+^ T cells into the kidneys after cisplatin treatment was significantly suppressed by 6-shogaol. These results suggest that 6-shogaol decreased the excessive production of cytokines and chemokines, and suppressed the infiltration of immune cells, resulting in an amelioration of cisplatin-induced inflammatory responses. Given that necroptosis promotes inflammatory responses through the leakage of cellular contents into the extracellular space [53], the suppression of inflammation by 6-shogaol may be, at least partially, due to its inhibitory effect on necroptosis.

## 4. Materials and Methods

### 4.1. Animals and Treatment

Animal experiments were performed in accordance with the Institutional Animal Care and Use Committee of the Daegu Catholic University Medical Center Approval number: DCIAFCR-200626-13-Y, approval date: 26 June 2020). Seven-week-old male C57BL/6N mice were acquired from HyoSung Science Inc. (Daegu, Korea) and kept at 20–24 °C and 55% humidity for 1 week. The mice were divided into three groups (*n* = 8 per group): control (Con), cisplatin (CP), and cisplatin plus 6-shogaol (CP + 6-SHO). The CP group and the CP + 6-SHO group were given a single intraperitoneal injection of cisplatin (20 mg/kg; Sigma-Aldrich, St. Louis, MO, USA). The CP + 6-SHO group was also given an intraperitoneal injection of 6-shogaol [20 mg/kg; dissolved in dimethyl sulfoxide (DMSO); Cayman Chemical, Ann Arbor, MI] daily for 3 consecutive days, starting from 1 h after cisplatin injection. The Con group and the CP group received intraperitoneal injections of an equal volume of DMSO daily for 3 consecutive days. All mice were sacrificed 72 h after a single dose of cisplatin. The doses of cisplatin and 6-shogaol were selected based on the results of previous studies [25,33].

### 4.2. Assessment of Renal Function

Serum creatinine and BUN levels were analyzed using a creatinine assay kit (BioAssay Systems, Hayward, CA, USA) and a BUN assay kit (Thermo Fisher Scientific, Waltham, MA, USA), respectively, according to the manufacturers’ protocols.

### 4.3. Histological Analysis and IHC Staining

Isolated kidney tissues were immediately fixed in 10% formalin and then dehydrated in graded series of ethanol. After dehydration, the tissues were cleared in xylene and embedded in paraffin. Thin sections were mounted on glass slides and stained with H&E or PAS. The severity of tubular injury was scored semiquantitatively by estimating the percentage of damaged area: 0, 0%; 1, ≤10%; 2, 11–25%; 3, 26–45%; 4, 46–75%; and 5, 76–100% [54,55]. Tubular injury was assessed in five arbitrarily chosen fields at ×400 magnification per kidney sample. For IHC staining, the sections were probed with a primary antibody and then incubated with a secondary antibody. The primary antibodies used for IHC staining were as follows: anti-NGAL (Santa Cruz Biotechnology, Santa Cruz, CA, USA), anti-KIM-1 (Abcam, Cambridge, UK), anti-4-HNE (Abcam, Cambridge, UK), anti-RIPK3 (Abcam, Cambridge, UK), anti-F4/80 (Santa Cruz Biotechnology, Santa Cruz, CA, USA), or anti-CD4 (Abcam, Cambridge, UK) antibodies. Images were visualized and captured using a confocal microscope (Nikon, Tokyo, Japan). The percentage of stained areas with anti-NGAL, anti-KIM-1, anti-4-HNE, or anti-RIPK3 antibodies were determined in five arbitrarily selected fields at ×400 magnification per kidney sample. The number of cells stained with anti-F4/80 or anti-CD4 antibody was counted in five arbitrarily chosen fields at ×600 magnification per kidney sample.

### 4.4. Western Blot Analysis

Total proteins were extracted from kidney tissues with a lysis buffer and then loaded onto gradient polyacrylamide gels (Bio-Rad Laboratories, Hercules, CA, USA). Separated proteins were transferred from gels to nitrocellulose membranes. The membranes were probed with primary antibodies against NOX4 (Novus Biologicals, Littleton, CO, USA), MnSOD (Abcam, Cambridge, UK), RIPK1 (Cell Signaling, Danvers, MA, USA), RIPK3 (Cell Signaling, Danvers, MA, USA), p-MLKL (Abcam, Cambridge, UK), MLKL (Cell Signaling, Danvers, MA, USA), and glyceraldehyde-3-phosphate dehydrogenase (GAPDH; Cell Signaling, Danvers, MA, USA), and then incubated with horseradish peroxidase-conjugated secondary antibodies. GAPDH was used as an internal control. The protein bands were visualized using enhanced chemiluminescence reagents (Thermo Fisher Scientific, Waltham, MA, USA). The signal intensities of the bands were measured using ImageJ software (National Institutes of Health, Bethesda, MD, USA).

### 4.5. Real-Time Reverse Transcription-Polymerase Chain Reaction (RT-PCR)

Total RNA was extracted from kidney samples using Trizol reagent. Reverse transcription was carried out using the iScript^TM^ cDNA Synthesis Kit (Bio-Rad Laboratories, Hercules, CA, USA) according to the manufacturer′s protocol. Real-time RT-PCR reactions were performed using specific primers (Table 1), the Thermal Cycler Dice Real Time System III (TaKaRa, Tokyo, Japan), and the Power SYBR Green PCR Master Mix (Thermo Fisher Scientific, Waltham, MA, USA). GAPDH was chosen as an internal reference.

### 4.6. TUNEL Assay

Apoptosis was assessed using a TUNEL assay kit (Roche Diagnostics, Indianapolis, IN, USA) according to the manufacturer′s protocol. For nuclear staining, 4′, 6-diamidino-2-phenylindole (DAPI) was used. The number of cells stained with TUNEL was counted in five randomly selected fields at ×400 magnification per kidney sample.

### 4.7. Measurement of Serum Cytokines

Serum levels of TNF-α and IL-6 were measured using standard quantitative sandwich ELISA kits (R&D Systems, Minneapolis, MN, USA) according to the manufacturer’s protocol.

### 4.8. Evaluation of Oxidative Stress

Renal MDA levels were measured using a colorimetric/fluorometric assay kit (Sigma-Aldrich, St. Louis, MO, USA) according to the manufacturer’s instructions. The GSH/GSSG ratio was assessed using the Glutathione Detection Kit (Enzo Life Sciences, Farmingdale, NY, USA) according to the manufacturer′s protocol.

### 4.9. Statistical Analysis

Data are presented as mean ± standard error of the mean (SEM). Differences between groups were analyzed with one-way analysis of variance (ANOVA) and Bonferroni’s post hoc tests. For all the analyses, *p* values less than 0.05 were considered statistically significant.

## 5. Conclusions

In conclusion, our data demonstrated the protective action of 6-shogaol against cisplatin-induced renal dysfunction and tubular injury. These effects are probably due to the suppression of oxidative stress, tubular cell death, and inflammation. Because it is also known that 6-shogaol exerts anti-tumor effects on various types of cancer cells [56,57], it may be a useful treatment option for AKI in cancer patients receiving cisplatin therapy.

## Figures and Tables

**Table 1:** List of primers used in this study.

Gene	Primer Sequence (5′→3′)	Accession No.
iNOS ^1^	Forward: CGAAACGCTTCACTTCCAA Reverse: TGAGCCTATATTGCTGTGGCT	NM_010927
COX-2 ^2^	Forward: AACCGCATTGCCTCTGAAT Reverse: CATGTTCCAGGAGGATGGAG	NM_011198
5-LOX ^3^	Forward: ATTGTTCCCATTGCCATCCAGCTCA Reverse: TCGTTCTCATAGTAGATGCTCACCA	NM_009662
NOX4 ^4^	Forward: GAACCCAAGTTCCAAGCTCATT Reverse: GGCACAAAGGTCCAGAAATCC	NM_015760
Catalase	Forward: CAAGTACAACGCTGAGAAGCCTAAG Reverse: CCCTTCGCAGCCATGTG	NM_009804
MnSOD ^5^	Forward: AACTCAGGTCGCTCTTCAGC Reverse: CTCCAGCAACTCTCCTTTGG	NM_013671
TNF-α ^6^	Forward: GACGTGGAACTGGCAGAAGAG Reverse: CCGCCTGGAGTTCTGGAA	NM_013693
IL-6 ^7^	Forward: CCAGAGATACAAAGAAATGATGG Reverse: ACTCCAGAAGACCAGAGGAAAT	NM_031168
MCP-1 ^8^	Forward: TAAAAACCTGGATCGGAACCAA Reverse: GCATTAGCTTCAGATTTACGGGT	NM_011333
CCL5 ^9^	Forward: ATATGGCTCGGACACCACTC Reverse: TCTTCTCTGGGTTGGCACACA	NM_013653
GAPDH ^10^	Forward: ACTCCACTCACGGCAAATTC Reverse: TCTCCATGGTGGTGAAGACA	NM_001289726

^1^ Inducuble nitric oxide synthase; ^2^ Cyclooxygenase-2; ^3^ 5-Lipoxygenase; ^4^ Nicotinamide adenine dinucleotide phosphate oxidase 4; ^5^ Manganase superoxide dismutase; ^6^ Tumor necrosis factor-α; ^7^ Interleukin-6; ^8^ Monocyte chemoattractant protein-1; ^9^ C-C motif chemokine ligand 5; ^10^ Glyceraldehyde-3-phosphate dehydrogenase.

## Data Availability

Data are contained within the article.

## Sample Availability

Not available.
